# Reconfigurable engineered motile semiconductor microparticles

**DOI:** 10.1038/s41467-018-04183-y

**Published:** 2018-05-03

**Authors:** Ugonna Ohiri, C. Wyatt Shields, Koohee Han, Talmage Tyler, Orlin D. Velev, Nan Jokerst

**Affiliations:** 1 NSF Research Triangle Materials Research Science and Engineering Center (MRSEC), Durham, NC 27708 USA; 2 Department of Electrical and Computer Engineering, Duke University, Durham, NC 27708 USA; 3 Department of Chemical and Biomolecular Engineering, North Carolina State University, Raleigh, NC 27695 USA

## Abstract

Locally energized particles form the basis for emerging classes of active matter. The design of active particles has led to their controlled locomotion and assembly. The next generation of particles should demonstrate robust control over their active assembly, disassembly, and reconfiguration. Here we introduce a class of semiconductor microparticles that can be comprehensively designed (in size, shape, electric polarizability, and patterned coatings) using standard microfabrication tools. These custom silicon particles draw energy from external electric fields to actively propel, while interacting hydrodynamically, and sequentially assemble and disassemble on demand. We show that a number of electrokinetic effects, such as dielectrophoresis, induced charge electrophoresis, and diode propulsion, can selectively power the microparticle motions and interactions. The ability to achieve on-demand locomotion, tractable fluid flows, synchronized motility, and reversible assembly using engineered silicon microparticles may enable advanced applications that include remotely powered microsensors, artificial muscles, reconfigurable neural networks and computational systems.

## Abstract (web summary)

Active particles that demonstrate life-like behavior may find use in bio-inspired technologies, but achieving on-demand reconfiguration remains challenging. Here, the authors demonstrate controllable, collective behavior in silicon microparticles, which are fabricated via conventional semiconductor methods.

## Introduction

A central goal of active matter research is to mimic the natural mechanisms of biological systems to create new bio-inspired technologies. One of the most captivating and complex biological systems is the brain due to its ability to form memories and store information through neural networks^1^. A distinctive feature of these networks is their plasticity, which entails the rapid formation and dissolution of neural links in response to stimuli^2^. Although much work has been done to assemble colloidal particles into structured networks^3–5^, such assemblies have been limited by their inability to controllably reconfigure, which enables the formation of new links. The use of intentionally reconfigurable active particles could overcome these challenges, as they can self-interact and form dynamic patterns^6–9^.

Active particles consume and dissipate energy to drive their motion, thus mimicking natural systems such as molecular motors, swarming bodies, and stimuli-responsive materials. The motion of active particles is strongly dependent upon the materials comprising them, their geometry, and the source of energy (which may be from, e.g., electric^10–13^, magnetic^14–18^, optical^19,20^, chemical^21–23^, and thermal origins)^24,25^, all of which govern their range, navigational control, and propulsion efficiency. Examples of active particles include nanoswimmers^26,27^, metallodielectric nanocylinders^28–30^, Janus spheres^31,32^, and asymmetric particle doublets^33–35^. Although these particles have been powered by different, and in some cases multiple mechanisms^36^, no particle has been shown to controllably assemble and disassemble on demand into plastically reconfigurable and electrically functional assemblies.

**Fig. 1:**
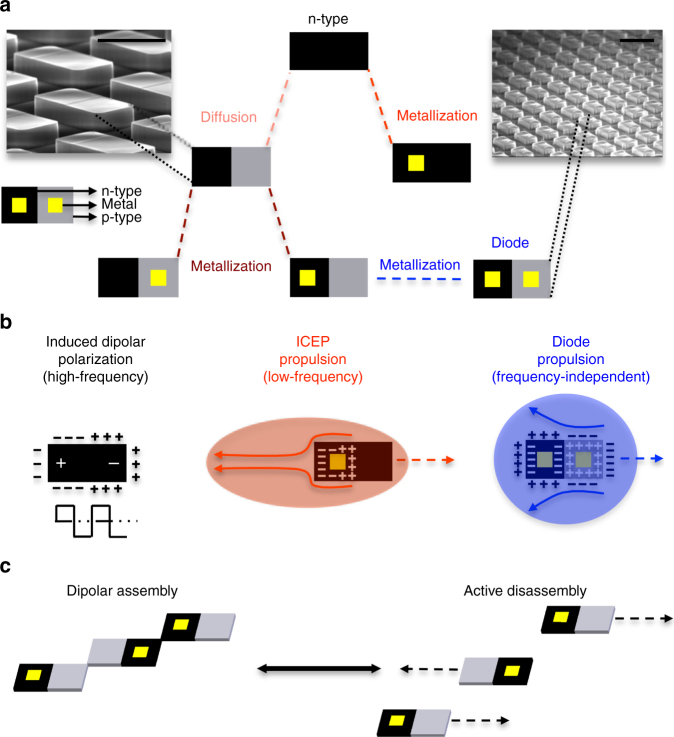
Semiconductor microparticle types and their controlled locomotion and interaction in AC electric fields. **a** Scanning electron microscope (SEM) images of silicon (Si) microparticles (scale bar = 20 μm) on bulk semiconductor substrates before separation (top corners) and schematics of different types of microparticles enabled by this integrated circuit fabrication approach (center). The black areas in the schematics represent Si with n-doped regions; the gray areas represent Si with p-doped regions; the yellow areas represent gold (Au) electrical contacts deposited on the microparticles. **b** Different types of semiconductor microparticles displaying three modes of programmed electrokinetic motion (from left to right): DEP, ICEP, and diode-based propulsion due to AC field rectification. In DEP, particles move due to induced dipoles formed from a non-uniform electric field at high frequencies. In ICEP, particles move due to the higher polarizability of one region of the particle (e.g., the Au contact) compared with other regions of the particle. In diode-based propulsion, particles move due to the rectifying capability of the internal circuit to direct current in one direction. **c** Reconfigurable assembly dynamics of active Si microparticles. At high frequencies (≥ 10 kHz), microparticles assemble by polarizing forces from DEP; at low frequencies (< 10 kHz), microparticles switch modes to disassemble and propel by ICEP

Given the intrinsic connection between particle design and function (or behavior)^37,38^, we have developed a class of active semiconductor and diode microparticles that can be comprehensively designed in size, shape, composition, and polarizability, as shown in Fig. 1a. This design capability can lead to control over the internal and external charge distributions, polarizabilities, and field rectification of the particles in AC electric fields. Thus, these particles can be engineered to draw energy to interact and propel in a variety of controllable fashions. We demonstrate their controllable motion through multiple concurrent mechanisms, including dielectrophoresis (DEP)^39^, induced charge electrophoresis (ICEP)^32^, and diode-based propulsion by AC field rectification (Fig. 1b)^12,13^. This combination of mechanisms enables a broad range of programmed collective behaviors (e.g., repulsion, bidirectional locomotion, microparticle chaining, and reversible assembly; Fig. 1c), which could enable their use in applications that include self-healing materials and reconfigurable networks.

## Results

### Design summary for thin film silicon microparticles

**Fig. 2:**
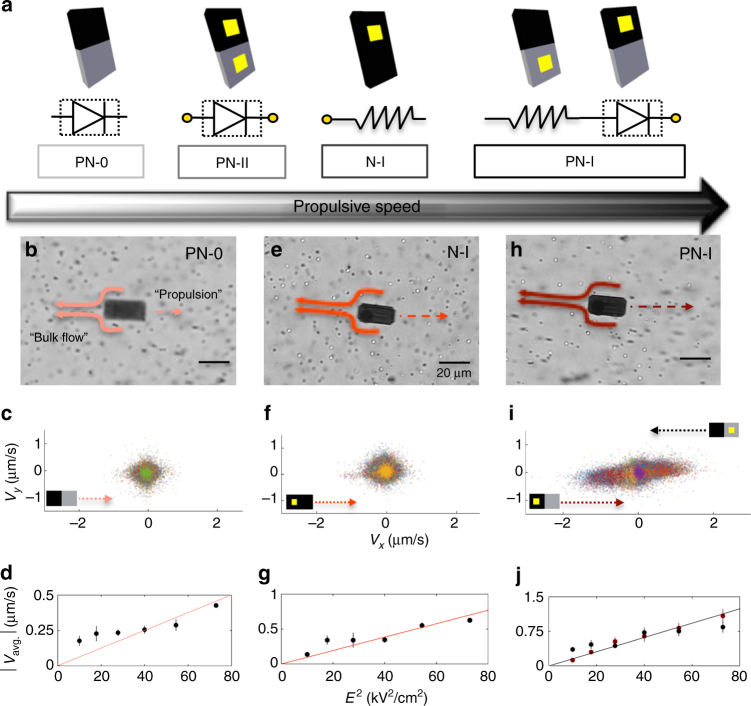
Hydrodynamic analyses of the flows driven by PN-0, N-I, and PN-I microparticles. **a** Electrical device representations and equivalent circuit diagrams of different types of microparticles (in order of propulsive speed): PN-0, PN-II, N-I, and PN-I. **b** Tracer beads flowing around a PN-0 microparticle at a fixed field strength (*E*^2^ = 54.4 kV^2^ cm^−2^) and frequency (100 Hz). **c** The *x*- and *y*-velocity of tracer beads surrounding a PN-0 microparticle, indicating slow fluid flows. **d** Velocity of PN-0 microparticles as a function of the square of the electric field, indicating that their motion arises from weak-ICEP forces from the p-n junction. **e** Tracer beads flowing around an N-I microparticle at a fixed field strength (*E*^2^ = 54.4 kV^2^ cm^−2^) and frequency (100 Hz). **f** The *x* and *y-*velocity of tracer beads surrounding an N-I microparticle, indicating fluid flows with an intermediate speed. **g** Velocity of N-I microparticles as a function of the square of the electric field, indicating motions are due to ICEP forces from the Au contact. **h** Tracer beads flowing around a PN-I microparticle at a fixed field strength (*E*^2^ = 54.4 kV^2^ cm^−2^) and frequency (500 Hz). **i** The *x* and *y*-velocity of tracer beads surrounding a PN-I microparticle, indicating fast fluid flows. **j** Velocity of PN-I microparticles as a function of the square of the electric field, indicating motions are due to strong-ICEP forces via combined interactions from the p–n junction and the Au contact. Note: the propulsion characterization of the PN-II particles (or diode microparticles) is shown in Fig. 5. Each data point represents the average and SD (one above and one below for the error bars), as measured from five different microparticles in a single experiment. Scale bar =  20 μm

In this study, six types of thin film semiconductor silicon microparticles were designed and fabricated (see the Methods section and Supplementary Notes 1 -  5 for more details on the fabrication process). The microparticles were fabricated on silicon-on-insulator (SOI) wafers, where the uppermost device layer (3.5 μm thick) consisted of n-type Si (*N*_D_ was approximately equal to 10^15^ cm^−3^) that served as the core material of the particles. This multi-layered wafer structure was fabricated based upon the desired behaviors of the different custom microparticles. Photolithography was used to define the geometry of the microparticles (e.g., shape and size) and other fabrication processes (e.g., diffusion, metallization, dry etching, and wet-etching techniques) were used to engineer and encode responses into the six different types of microparticles. The following nomenclature was developed to describe the different types of microparticles (Fig. 2a; in order of propulsion speeds): PN-0 is a microparticle with a p–n junction and without metal contacts; PN-II is a diode microparticle with a p–n junction and with metal contacts on both the n-side and the p-side; N-I is an n-type silicon microparticle with a metal contact on one side; and PN-I is a microparticle with a p–n junction and with one metal contact on either the n-side or on the p-side. Although our particles were 10 μm × 20 μm (surface area), we note that this approach can be adapted to make particles of smaller or larger size. Traditional top-down photolithography can create well-defined features as small as 0.5 μm, and electron-beam lithography can be used to pattern particles and coating features as small as 50 nm^40^.

A key advantage of this integrated circuit fabrication approach is the ability to diffuse patterned regions of the microparticles with dopants. For the case of the PN-0, PN-II, and PN-I microparticles, we altered the electrical properties of the silicon material by patterning and diffusing specific regions with dopants to attain p-type compositions on half of the microparticle. Thus, we formed p–n junctions, which enabled distinct differences in their behaviors when stimulated by AC electric fields. The two metal contacts on the PN-II microparticle enabled internal rectifying flow of electronic carriers (electrons and holes) across the p–n junction (see Supplementary Note 5 for details), and was the only p–n junction particle capable of this internal flow. We also note that pure n-type microparticles were fabricated with uniform doping and without metallization (N-0; see Supplementary Note 1 for more details on the fabrication of the N-0 microparticle).

Once fabricated, the microparticles were released from the silicon substrate by sacrificially etching the silicon dioxide layer (SiO_2_), washed to remove impurities, and suspended in deionized (Millipore) water for experimental testing (see Supplementary Note 1 for details on microparticle preparation). We note that as soon as the microparticles are exposed to water, a thin SiO_2_ typically forms over exposed Si surfaces^41,42^. Each type of microparticle was subjected to an external AC electric field generated in a fluidic chamber with co-planar gold electrodes (electrical circuit equivalent shown in Fig. 2a). The independent variables of the electric field, *E*, used to power the microparticles include the field strength (*E*^2^ was approximately equal to 10–75 kV^2^ cm^−2^) and AC frequency (0.05–500 kHz; see Supplementary Note 7 for detailed field and frequency-based tracking analyses of the fabricated microparticles). In all cases, the microparticles initially aligned with their longest axis parallel to the electric field due to dielectrophoretic torque^43^.

### Hydrodynamic flow analysis of PN-0, N-I, and PN-I microparticles

First, we performed a series of tracer experiments (*E*^2^ = 54.4 kV^2^ cm^−2^ and 500 Hz; Supplementary Movie 1 through Supplementary Movie 5) to understand the relationship between microparticle design and the mechanism of propulsion for the PN-0 (Fig. 2b-d), N-I (Fig. 2e-g), and PN-I (Fig. 2h-j) particles. Before the start of each experiment, 500 nm latex beads were suspended in the chamber and became randomly dispersed around the microparticles. We used ImageJ (NIH) to quantify the speed of tracer bead movement and thus localized fluid flow. In separate experiments without tracer beads, we tracked the speed of the semiconductor microparticles in response to stimulation from AC electric fields (*E*^2^ = 54.4 kV^2^ cm^−2^ and 500 Hz). We found that, of these particles, the PN-0 microparticles had the slowest propulsion velocities (note that N-0 is reported in the SI and had the slowest propulsion velocity of all particles studied) and the PN-I microparticles had the fastest velocities.

For the case of the PN-0 microparticles, the tracer beads revealed a slow flow toward the p–n junction along the sides of the microparticle (Fig. 2b). In Supplementary Movie 1, the tracer beads are seen to flow with an average speed of 250 nm s^−1^ in the *x*-direction (note that the PN-0 microparticle propels with the p-side facing forward (described below); tracer beads predominantly move in the opposite direction; Fig. 2c). As is common for second-order propulsive effects such as ICEP, the velocity of the PN-0 microparticles was found to be dependent on the square of the electric field *E*^2^, $U_{\mathrm{ICEP}} \propto E^2$ (Fig. 2d). Weak anisotropic polarization (weak-ICEP) directionally propelled the microparticles through the fluid where the electrical conductivity of the doped n-type silicon, with phosphorous atoms (*σ* is approximately equal to 1 × 10^5^ S cm^−1^), is higher than that of the doped p-type silicon, with boron atoms (*σ* is approximately equal to 1 × 10^−6^ S cm^−1^)^44^. This effect is caused by the non-uniform dopant concentration across the p–n junction of the microparticle where the n-side acts as the propeller and the p-side acts as an insulating coating^45^. In this case, the n-side of the microparticle had a uniform dopant concentration (*N*_D_) of ~ 10^15^ cm^−3^. The p-side of the microparticle, which was an n-type material counter-doped with p-type boron spin-on diffusants, had a maximum dopant concentration (*N*_A_) of ~ 10^20^ cm^−3^ at the surface. This non-uniform doping concentration creates a non-uniform charge distribution of counterions in the surrounding fluid^41,42^, causing weak-ICEP flows and propulsion of the PN-0 microparticles when exposed to an AC electric field. To understand the relationship between the doping concentration, counterionic charge distribution, and polarizability, we also evaluated the surface potential for each silicon microparticle composition (in water) and found that the magnitude of the potential was in qualitative correlation to the particle propulsive speeds (see Supplementary Table 1). The most likely reason for this correlation is that particles with uniform doping (N-0) had a lower estimated surface potential than particles with non-uniform doping (PN-0). Particles with a metal contact (N-I and PN-I) had a higher estimated surface potential than particles with uniform doping (N-0) or particles that were symmetrically polarized (PN-II).

For the case of the N-I microparticles, the tracer beads revealed a flow toward the metal contact along the sides of the microparticle (Fig. 2e). In Supplementary Movie 2, the tracer beads are seen to flow at an average speed of 300 nm s^*-*1^ in the *x*-direction (note that the N-I microparticle propels with the non-metal side facing forward; as tracer beads predominately move in the opposite direction; Fig. 2f). As expected, the average velocity of the N-I microparticle was closely proportional to the square of the electric field (at a fixed frequency of 100 Hz; Fig. 2g)^32^. Supplementary Movie 3 shows two N-I microparticles propelling by the ICEP effect (from the metal) at 500 Hz. Here, the presence of the metal contact enabled strong anisotropic polarizations that actively propelled the particles through the fluid. This phenomenon was first observed with metallo-dielectric Janus particles^32^ and was studied in various asymmetric systems of polarized particles and electrodes^46–48^. It has been shown that motion by ICEP can be achieved in the following frequency regime, *D* *k*^−1^ *L*^−1^ ≤ *w* *≤* *D* *k*^−1^ *a*^−1^, where *k* is the Debye length, *D* is the ion diffusion coefficient, 2 *L* is the electrode separation, and *a* is the particle size^32^. This propulsion originates from the non-linear distribution of the ionic double layer induced around the microparticles^32^. Here, the electrical conductivity of the metal (*σ* is approximately equal to 1.4 × 10^8^ S cm^−1^)^49^ is orders of magnitude higher than that of the n-type silicon (*σ* is approximately equal to 2.7 × 10^−1^ S cm^−1^)^50^. This asymmetric induced charge distribution generates macroscopic ion-driven fluid flows that drive microparticle propulsion in a direction opposite to the metal contact^51^. The slight nonlinearities in the velocity of the N-I microparticle can be attributed to the charge build-up from the metal-semiconductor junction interface when a highly polarizable metal (Ti/Ni/Au) is in contact with a low doped n-type semiconductor material (10^15^ cm^−3^).

For the case of the PN-I microparticles, the tracer beads rapidly flowed toward the p–n junction and the metal contact from the sides of the microparticles (Fig. 2h). In Supplementary Movie 4, the tracer beads are seen to flow at an average speed of 750 nm s^−1^ in the *x*-direction (note that the PN-I microparticle propels with the non-metal side facing forward; tracer beads predominately move in the opposite direction; Fig. 2i). This relatively high fluid velocity generated by the PN-I microparticle is due to the presence of the combined p–n junction and highly polarizable metal. Supplementary Movie 5 shows several PN-I microparticles propelling by the combination of the p-n junction and the ICEP effect (from the metal) at 500 Hz. As expected, the average PN-I microparticle velocity was proportional to the square of the electric field (at a fixed frequency of 500 Hz; Fig. 2j). Tracer experiments and polarization flow origins for the remaining types of microparticles (i.e., N-0 and PN-II) are discussed in Supplementary Note 8.

**Fig. 3:**
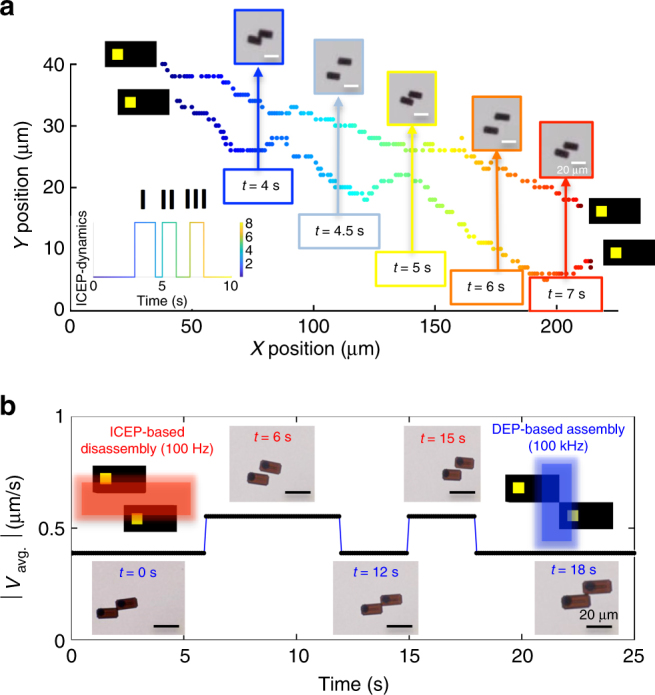
Synchronized rebounding (cyclic oscillations from combined attraction and repulsion) and reversible assembly of N-I microparticles. **a** Trajectories of rebounding N-I microparticles (scale bar = 20 μm), plotted in Cartesian coordinates, are shown at a fixed electric field strength (*E*^2^ = 54.4 kV^2^ cm^−2^) and frequency (100 Hz). The graphical inset also illustrates the synchronized rebounding motion of the N-I microparticles. The high pulses (I, II, and III) represent regions where the N-I microparticles are deflected off of each other. The low pulses represent regions where the N-I microparticles propel through the fluid. These dynamics are shown in real time in Supplementary Movie 6 and Supplementary Movie 7. **b** Micrographs of two N-I microparticles (scale bar = 20 μm) assembled corner-to-corner at high frequencies (100 kHz; *E*^2^ = 27.8 kV^2^ cm^−2^). The two microparticles reversibly and repeatedly disassemble and propel in parallel at low AC field frequencies (100 Hz). These dynamics are shown in real time in Supplementary Movie 9

Overall, these results indicate that the p–n junction, metal contact, and the interface between the two materials contribute to the active propulsion of microparticles via ICEP, with the metal contact being more polarizable, as it drives much larger fluid flows. This makes it possible to program different modes of active electrical propulsion simply by changing the design of the microparticles. Importantly, as we discuss next, we can also control the fluid flows around the microparticles simply by changing the signal of the AC electric field, which can lead to direct control over the collective interactions between microparticles. Specifically, we show that we can electrically program the microparticles to: (i) continuously rebound in a controlled fashion (Fig. 3a), and (ii) controllably assemble and disassemble on demand by switching the frequency of the external electric field (Fig. 3b).

### Cyclic attraction, repulsion, and reversible assembly of N-I microparticles

By making use of the well-controlled fluid flows around the N-I microparticles (Fig. 2e-g), we demonstrate the ability of a pair of microparticles to synchronously rebound (cyclically attract and repel; Fig. 3a). In Supplementary Movie 6 and Supplementary Movie 7 (100 Hz), two N-I microparticles propel in the same direction when sharing the same orientation (i.e., when the two metal contacts are aligned side-by-side). In this case, cyclic rebounding occurred as the particles drew closer around 4, 5, and 7 s. The N-I microparticles propelled by ICEP^32^ and approached each other due to long-range dipolar attraction forces^31^. However, the rebounding occurred due to the overlap of the hydrodynamic flows (at low AC frequencies) driven by the double layer ionic charges, in the moving liquid, around each microparticle (Fig. 2e-g). This collective swimming effect is a result of the asymmetric design of the N-I microparticles. We are not aware of previous reports demonstrating this type of synchronized swimming for diode microparticles and expect that in the future, it may be possible to design systems that take advantage of such synchronized swimming-rebounding dynamics. For example, Supplementary Movie 8 shows PN-0 microparticles also hydrodynamically rebounding, at low AC frequencies (100 Hz), by weak-ICEP polarization forces. In this case, cyclic rebounding occurs due to the overlap of the hydrodynamic flows and when the microparticles share the same orientation (i.e., when the n-side of the particle are aligned side-by-side).

**Fig. 4:**
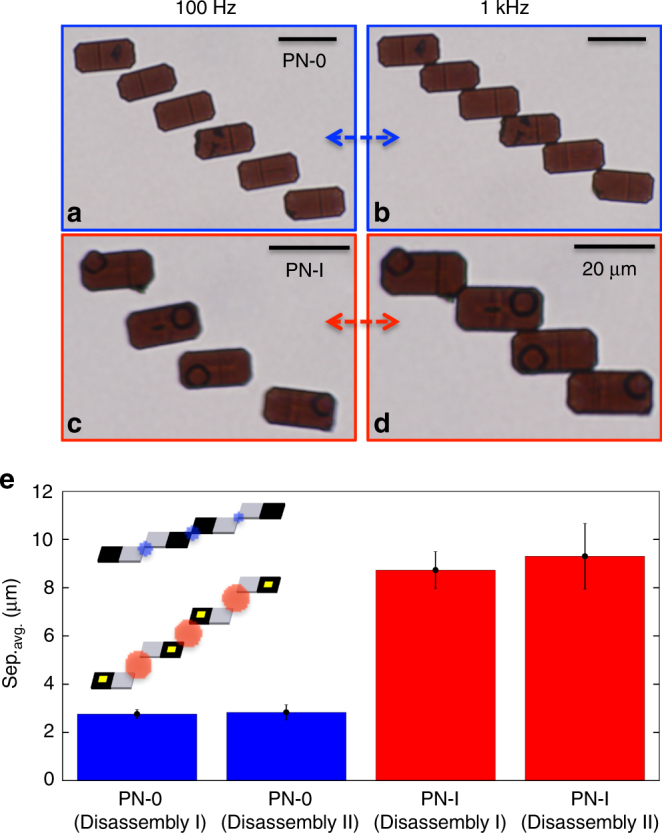
Electrically switchable assembly and disassembly of PN-0 and PN-I microparticles. **a**–**d** Interaction of six PN-0 (**a**, **b**) and four PN-I (**c**, **d**) microparticles (scale bar = 20 μm) at frequencies of 100 Hz (left) and 1 kHz (right) at fixed field strengths of *E*^2^ = 40 kV^2^ cm^−2^. **e** Average separation distance between microparticles. The blue bars correspond to the average distance between PN-0 microparticles 1.0 s after disassembly (at *t*_1_ = 6.0 and *t*_2_ = 12.0 s). The red bars correspond to the average distance between PN-I microparticles 1.0 s after disassembly (*t*_1_ = 6.0 and *t*_2_ = 12.0 s). The error bars represent SD (one above and one below for the error bars) for measurements between multiple disassembly events in a single experiment

To investigate the effect of frequency modulation on microparticle interactions and dynamics, we analyzed the reversible assembly of two N-I particles by switching the frequency of an AC electric field (Fig. 3b). The particles actively propelled at low frequencies (< 10 kHz) via ICEP and assembled into a short, staggered chain at high frequencies (≥ 10 kHz) via DEP. From *t* = 0.0–6.0 s (low frequencies), the microparticles separated due to the decrease in the polarizability of the ionic layer, from the metallic patches on the surface of the particles. Electrokinetically, the electric double layer has sufficient time to charge in this frequency regime and generate ionic flow that powers the microparticle to propel through the fluid. Physically, the particles separated and synchronously propelled due to the overlap of the hydrodynamic flows driven by the double layer charges in the moving liquid around each similarly oriented microparticle. The active propulsion of the N-I microparticles during their disassembled state can thus enable rapid mixing and rearrangement, which would otherwise be slow given the near-negligible diffusivity rate of the microparticles due to their size. However, from *t* = 6.0–12.0 s, a high-frequency electric signal was applied and ICEP was suppressed, thus allowing DEP to drive the microparticles to assemble. At 100 kHz, the mobility of the induced charges around the microparticles is suppressed, and thus they assemble spontaneously by dipolar attraction^43,52,53^. Interestingly, these two dynamical modes of attraction and repulsion are completely reversible. This process is shown in real time (Supplementary Movie 9) and is investigated more in detail later (Fig. 4).

### Collective reversible assembly and disassembly of PN-0 and PN-I microparticles

Next, we investigated the switchable assembly and disassembly of ensembles of two types of microparticles (PN-0 and PN-I; Fig. 4). For the case of PN-0 microparticles, weak anisotropic polarization (weak-ICEP) forces propelled the particles through the fluid, as described above (Fig. 2b-d). As seen in Supplementary Movie 10 and Supplementary Movie 11 (at *E*^2^ = 40 kV^2^ cm^−2^), the microparticles propelled with the p-side facing forward, as expected. At low frequencies (Supplementary Movie 12; 100 Hz; Fig. 4a), the PN-0 microparticles disassembled and propelled via weak anisotropic polarization forces from the non-uniform dopant distribution across the p–n junction. At higher frequencies (Supplementary Movie 12; 1 kHz; Fig. 4b), the PN-0 microparticles assembled into a staggered chain via dipolar polarization attraction. In particular, long-range dipolar interactions dominate when the PN-0 microparticles are spaced several micrometers from each other (e.g., > 10 μm), whereby microparticles still propel with the p-side facing forward. When the PN-0 microparticles are spaced only a few micrometers from each other (e.g., < 10 μm), short-range dipolar interactions begin to dominate and induce the microparticles to form chains. The PN-I microparticles also disassembled and propelled at low frequencies (Supplementary Movie 13; 100 Hz; Fig. 4c) via strong anisotropic polarization forces from the combination of the highly polarizable metal and weak non-uniform doping across the p–n junction (Fig. 2g-i). At higher AC electric field frequencies (Supplementary Movie 13; 1 kHz), the PN-I microparticles assembled into well-organized staggered chains due to strong dipolar polarization forces (Fig. 4d).

To better understand the relationship between microparticle type and behavior, we measured the separation distance between PN-0 and PN-I microparticles during their active disassembly process (100 Hz; Fig. 4e). After a fixed period of time, from reducing the frequency to 100 Hz (1.0 s for PN-0 and 1.0 s for PN-I), the PN-0 and PN-I microparticles had an average separation distance of 2.8 and 9.0 μm, respectively (*p* < 0.05), which corroborates our findings from the hydrodynamic flows around the microparticles (Fig. 2). The anisotropic polarization forces from the non-uniform doping across the p–n junction caused the PN-0 microparticles to weakly repel, whereas the anisotropic polarization forces from the metal contact caused the PN-I microparticles to strongly repel. To further study the relationship between different propulsive mechanisms involved during the disassembly process, tracer experiments were performed on PN-0 and PN-I microparticles (see Supplementary Movie 18 and Supplementary Movie 19 performed at *E*^2^ = 40 kV^2^ cm^−2^, and Supplementary Note 9 for details).

We also studied the dynamics of higher concentrations of PN-I microparticles (i.e., greater than 100 particles in a single microscope frame; see Supplementary Movie 14, Supplementary Movie 15, Supplementary Movie 16, and Supplementary Note 10 for details). These microparticles also statically assembled and disassembled around the same frequency regimes as for the N-I particles (Fig. 3b), but their separation distances during disassembly varied (average separation distance for N-I microparticle was 5.4 μm after 1.0 s) as a function of particle composition (Fig. 4e). These data corroborate our findings from the hydrodynamic flows and variation in propulsive effects depending on microparticle composition (Fig. 2). Further, these particles assembled into straight chains at frequencies higher than those shown in Fig. 4 (i.e., 10 kHz), which was not reversible, unlike the staggered chains formed by PN-0 and PN-I microparticles.

### Self-propulsion of PN-II diode microparticles

**Fig. 5:**
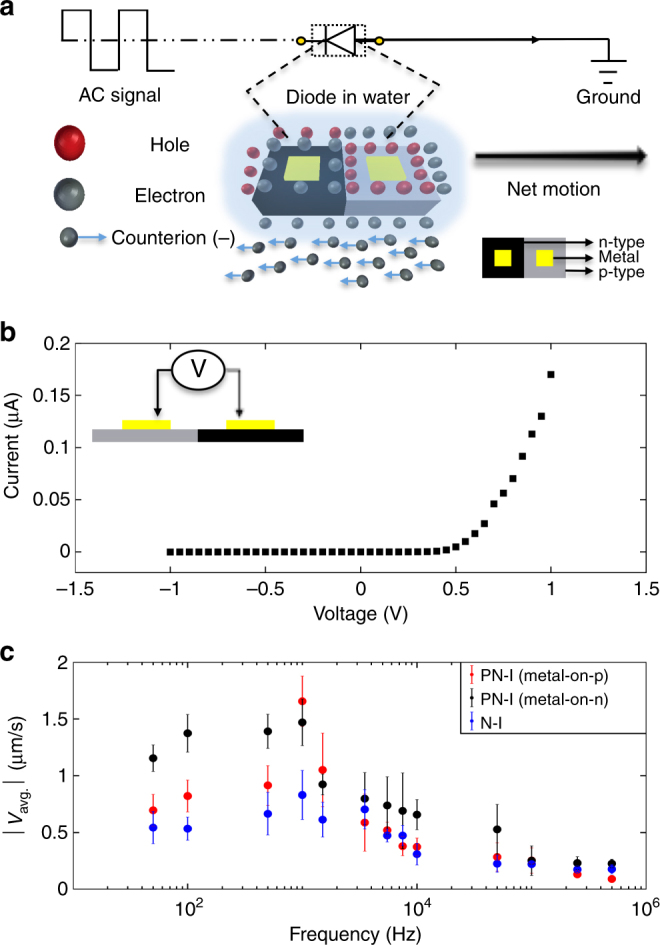
Electrical propulsion and frequency analysis of p–n junction and p–n junction diode microparticles. **a** Electrical circuit diagram of a p–n junction diode microparticle (PN-II) suspended in water. An AC electric field is applied to a fluidic cell containing gold electrodes. Large red and grey spheres represent positively and negatively charged electronic carriers, respectively, which can diffuse across the diode microparticles; small gray spheres with blue arrows represent negative counterions in the fluid. **b** Current–-voltage (*I*–*V*) characteristic curve of PN-II diode microparticles. The *I*–*V* curve was measured with a probe on each side of the p–n junction diode (inset). **c** Frequency analysis of the two types of PN-I microparticles (black and red for p–n junction microparticles with the metal contact on the n-side and p-side, respectively) in comparison to the N-I microparticles (blue). Data were collected at a fixed *E*^2^ = 54.4 kV^2^ cm^−2^. Each data point represents the average and SD (one above and one below for the error bars), as measured from five different microparticles in a single experiment

Finally, we investigated PN-II diode microparticles, which are the only electrically active diodes in this study due to the presence of metallic contacts on each side of the p-n junction. The electrical diode characteristics of the PN-II diode microparticle, was verified by measuring their *I*–*V* (current–voltage) curves on the SOI substrate before lateral etching (Fig. 5b). Similar to the PN-0, N-I, and PN-I microparticles, the PN-II microparticles propelled through the fluid in response to an AC electric field. As established previously, the particles are polarized only in the positive half of the cycle, and rectified the AC electric field into a local DC potential^12,13^. This DC potential directionally propels the microparticles by electro-osmotic flows generated around their surfaces (Fig. 5a; see Supplementary Movie 17 performed at *E*^2^ = 40 kV^2^ cm^−2^ to understand how the microparticles hydrodynamically propel by self-electroosmotic flows)^12,13^.

Once released from the substrate and exposed to an AC electric field (at a fixed field strength of *E*^2^ = 54.4 kV^2^ cm^−2^), the tracking analysis revealed significant differences in the velocity of the asymmetrically polarized PN-I microparticles and the asymmetrically polarized N-I microparticles (Fig. 5c; note that this relationship was also examined for microparticles (e.g., PN-II); see Supplementary Note 7 for details). Both types of PN-I microparticles exhibited a strong frequency-dependent propulsion behavior. The particles attained higher speeds than the PN-II diode microparticles at low frequencies (< 10 kHz), but were slower at high frequencies (≥ 10 kHz). In contrast, the PN-II diode microparticles demonstrated a frequency-independent propulsion behavior different from all other compositions. Specifically, as the frequency increased above 10 kHz, the particles maintained their motion at nearly constant velocity. This finding reveals that strong-ICEP effects (from the metal contacts) dominate the motion of microparticles with one metal contact at low frequencies (i.e., < 10 kHz), which does not occur with the PN-II diode microparticles. Further, these results show that the diode microparticles are able to rectify the AC signal at frequencies above 10 kHz, which enables another level of control and may be used for designing semiconductor microdevices that actively propel even when energy is provided at ultrahigh (e.g., radio) frequencies.

## Discussion

We report a class of custom semiconductor microparticles that are comprehensively designed to draw energy from AC electric fields to actively propel, assemble, dissemble, and reconfigure on demand. We show that this approach enables multiple modes of propulsion (driven by, i.e., DEP, ICEP, and diode rectification currents), which result in multiple deterministic collective interactions. We further reveal how the collective dynamic behavior of small ensembles of motile particles can be tuned by changing the frequency of the electric field. This demonstration shows silicon microparticles that can switch between multiple modes of active locomotion, while also being able to move in long-range coordinated cyclic manner and driven to rapidly assemble and disassemble on demand. This capability may give rise to systems that can form structured networks and liquefy to reconfigure into new structures, toward plastically deformable electronic circuits and synthetic neural networks^2^. This level of “knob-controlled” dynamics provides a step forward in realizing the synthetic expression of natural systems such as atomic freezing and melting^54^, as well as the formation of percolated gels^55^.

We believe that understanding the physical characteristics of the semiconductor-oxide-liquid and the semiconductor-metal-oxide-liquid interfaces is critical to the design of new types of active semiconductor microparticles (see Supplementary Note 11 for details on the electrostatic potential across these key interfaces)^41,42,56^. The distribution of electronic charges in the semiconductor, the semiconductor-oxide interface, and the interaction between the oxide and metal surfaces engenders ionic charges in the surrounding liquid, which directly affects the polarizability and propulsion of the microparticles when subjected to AC electric fields. The doping profile of the microparticles has a significant effect on the formation of surface charges and on the electrostatic potential distribution in the fluid surrounding the microparticles, which thus affects the distribution of ions in the fluid and the multipolar interaction between groups of microparticles^41,42,56^. Further, the choice of metal (e.g., ohmic or Schottky) could lead to very interesting behaviors and properties at the semiconductor-metal junction interface. Thus, the ability to engineer the interfacial (both internal and external) electrostatic properties of the semiconductor^41,42,56^, along with their size, shape, and doping, is critical to the design of future motile assembling and self-reconfiguring microcircuits.

This study provides the groundwork for understanding the relationship between the design of semiconductor microparticles and their response to AC electric fields, which includes three phenomenologically distinct locomotive effects that lead to a wide variety of controllable interactions and switchable dynamics. We anticipate that further alterations in the geometry (e.g., shape and size), electrical properties (e.g., patterned dopant diffusion and metallization), insulating properties (e.g., integrating dielectric patterns), and applied field effects (e.g., electrical, and additionally, optical and magnetic) will result in higher-level functionalities that enable their use as a class of highly programmable active matter towards new types of electromechanical switches, artificial muscles, dynamically reconfigurable circuitry, dynamically reassembled bio-inspired neural networks, and other intriguing applications^57–59^.

## Methods

### Cleanroom fabrication

Microparticles were fabricated in the Shared Materials Instrumentation Cleanroom Facility (SMiF) at Duke University (see Supplementary Note 1 for further details on the thin film silicon fabrication process). Briefly, the microparticles were defined on SOI wafers ((100) orientation), which consisted of a 3.5 μm-thick n-type phosphorous-doped Si device layer (calculated background concentration of device layer (*n*_device_ = 7.22 × 10^14^ cm^−3^, *ρ*_device_ ~ 6.17 Ω cm, University Wafer, Inc.), bonded to a 2 μm-thick buried SiO_2_ (BOX) layer, bonded to a 525 μm-thick n-type antimony-doped Si handle substrate.

The thin film fabrication process began with a three-part standard RCA clean to remove organic contaminants (by 10 min of submersion in 5:1:1 H_2_O:H_2_O_2_:NH_4_OH), thin native oxide layers (by 5 s of submersion in a buffered oxide etchant (BOE)) and ionic contaminants (by 10 min of submersion in 6:1:1 H_2_O:H_2_O_2_:HCl) on the surface of the device layer. The next step in the fabrication process depended upon the target structure and intended functionality of the particles. To fabricate particles with p–n junctions, a 150 nm-thick layer of SiO_2_ was grown on the surface of the device layer by heating the wafer to 1000 °C for 5 h in a high-temperature furnace (Tempress 6304 4-stack O_2_ atmosphere furnace), which served as a diffusion mask for formation of the p-n junction. Next, the thermal oxide was patterned using topside photolithography (Karl Suss Mask Aligner MA6) and BOE was used to open the diffusion windows. The surface of the wafer was then coated with a boron-doped spin-on-glass (SOG, borosilicate film, surface concentration, *C*_surface_ = 6.57 × 10^20^ cm^−3^; Filmtronics, Inc.) to counter dope p-type regions into the device layer. The SOG was annealed at 1050 °C for 17.5 h in a high-temperature furnace (Tempress 6304 4-stack N_2_ and O_2_ atmosphere furnace) to achieve an estimated 3.5 μm junction diffusion depth into the particle mesas. Once the annealing process was complete, the residual dopant oxide was removed using a dry (Trion Technology Phantom II Reactive Ion Etcher (RIE)) and wet-etch process (i.e., BOE).

Next, an array of 4 μm × 4 μm square metal contacts was aligned (Karl Suss Mask Aligner MA6), patterned (JSR Micro NFR Negative Photoresist Series) using topside photolithography, ashed in an O_2_ plasma (Emitech K-1050X), vacuum deposited (Kurt Lesker PVD 75 Electron Beam Evaporator), and annealed (Jipelec JetFirst 100 RTA) using a rapid thermal annealer. Each of these steps were completed on each side of the p-n junction. A Titanium (Ti; 800 Å)–Nickel (Ni; 600 Å)–Gold (Au; 2000 Å) metal stack was deposited on the n-side of the p–n junction. An Aluminum (Al; 600 Å)–Titanium (Ti; 500 Å)–Nickel (Ni; 400 Å)–Gold (Au; 1900 Å) metal stack was deposited on the p-side of the p–n junction. Both metal contacts were annealed (Jipelec JetFirst 100 RTA) at 350 °C for 10 s. The final step in the fabrication process was defining the rectangular mesas using topside photolithography (Karl Suss Mask Aligner MA6) followed by deep reaction ion etching (SPTS Pegasus Deep RIE; DRIE). The depth generated by the DRIE process was measured using a profilometer (Bruker Dektak 150) between the thin film silicon mesas. Next, the particles were contained in a solution of BOE (over a VWR® Scientific Standard Orbital Shaker, Model 5000) to selectively etch the BOX layer and to sacrificially release the mesas from the SOI substrate (see Supplementary Note 2, Supplementary Note 3, Supplementary Note 4, and Supplementary Note 5 for further details on the sacrificial release process). A small volume of microparticles, released in suspension, was extracted with a calibrated pipette. The sample was then washed three times (i.e., by centrifuging 2000 × *g* for 2 min, VWR® Scientific, decanting the supernatant, and suspending the pellet in fresh Millipore water). Finally, the sample was placed in a testing chamber for experimental testing (see details on the experimental chamber and testing below).

### Characterization

A representative set of devices (still attached to the substrate) was electrically probed using a source measurement unit (SMU; Keithley Instruments, Co.) to measure the *I*–*V* characteristic curve (see Supplementary Note 6 for further details on the characterization process for the microparticles). A 200 nm diameter, 2”-long tungsten cat whisker tip (Lucas Signatone, Corp.) was used to probe the metal electrodes. A DC voltage sweep was set up across the gold electrodes for characterization.

### Experimental chamber and testing

The experimental chamber was constructed on a glass slide where two co-planar gold electrodes (10 cm long × 1 cm wide with a 3 mm inter-electrode gap) were vacuum deposited by evaporating 100 Å of Cr followed by 1000 Å of Au. A 20 μL suspension of the microparticles was placed into a corral secured by a 100 μm tall imaging spacer (Grace Bio-Labs, Inc.) on the glass slide (VWR® Scientific). A coverslip glass (VWR® Scientific) was placed on top of the spacer, thus forming a thin experimental cell between the glass slide and the coverslip. The droplet spread throughout the inside of the imaging spacer, resulting in contact with both electrodes. Copper connective wires were attached to the gold electrodes in the testing chamber. An AC electric field was supplied to the electrodes through the copper wires by a function generator (33120 A, Agilent Technologies, Inc.) connected to a power amplifier (PZD 700, Trek, Inc.). All experiments were performed under a bright-field optical microscope (Olympus SZ-61).

### Data availability

The data that support the findings of this study are available from the authors upon request.

## Electronic supplementary material

Supplementary Information

Description of Additional Supplementary Files

Supplementary Movie 1

Supplementary Movie 2

Supplementary Movie 3

Supplementary Movie 4

Supplementary Movie 5

Supplementary Movie 6

Supplementary Movie 7

Supplementary Movie 8

Supplementary Movie 9

Supplementary Movie 10

Supplementary Movie 11

Supplementary Movie 12

Supplementary Movie 13

Supplementary Movie 14

Supplementary Movie 15

Supplementary Movie 16

Supplementary Movie 17

Supplementary Movie 18

Supplementary Movie 19

Supplementary Movie 20
