# Supplementary material for: Reconfigurable engineered motile semiconductor microparticles
Source: Nat Commun. 2018 May 3;9:1791. doi: 10.1038/s41467-018-04183-y (PMC5934469; doi:10.1038/s41467-018-04183-y)
Supplement: Supplementary file 1 — Supplementary Information [file 41467_2018_4183_MOESM1_ESM.pdf]

Reconfigurable Engineered Motile Semiconductor Microparticles

Ohiri et al.

Supplementary Information

Supplementary Note 1: Fabrication of n-type silicon microparticles, p-n junction silicon microparticles, and p-n junction silicon diode microparticles

All of the silicon (Si) microparticles were fabricated in the Shared Materials Instrumentation Cleanroom Facility (SMiF) at Duke University. The material used to fabricate the microparticles were (100) orientation n-type silicon on insulator (SOI) wafers. The SOI wafers were comprised of: n-type Si (3.5 μm thick, phosphorous-doped) device layer; silicon dioxide (SiO_2; 2 μm thick, also referred to as a buried oxide, or BOX layer); n-type Si (525 μm thick, antimony-doped) handle substrate. The background doping concentration of the device layer was specified by the vendor to be ρ = 10 Ω-cm (UniversityWafer, Inc.). The background resistivity and doping concentration of the device layer was characterized after SOI purchase by a four point probe resisitivity measurement which yielded a resistivity of ρ = 6.17 Ω-cm, which corresponded to a donor doping concentration of $N_D = 7.22 \times 10^{14}$ cm^{-3}.

The shape, size, and metallic patterns on the microparticles were defined by photomasks, which were designed in AutoCAD® (Autodesk, Inc.) and printed by a photomask manufacturer (Photo Sciences, Inc.). The masks were chrome patterns on soda lime glass, and included fiducial alignment markers to ensure that the sequential process steps, which defined the particle, dopant layer, and metal contact patterns, were correctly aligned on the microparticles.

The thin film fabrication process began with cleaving a 4'' SOI wafer into 1 cm x 1 cm pieces, followed by a three-part standard Radio Corporation of America (RCA) clean to remove: (i) organic contaminants (5:1:1 H_2O:H_2O_2:NH_4OH, 10 minutes), (ii) thin native oxide layers (submergence in buffered oxide etchant (BOE), 10 sec) and (iii) any

ionic contaminates (6:1:1 H_2O:H_2O_2:HCl, 10 min) on the surface of the silicon device layer. The next step depended upon which type of microparticle was being fabricated.

Supplementary Note 2: Fabrication and sacrificial release of n-type microparticles without a metal contact (N-0)

For the n-type microparticles without a metal contact (N-0), individual rectangular mesas were patterned using topside positive photolithography (Microposit® S1813® Positive Photoresist) and a mask aligner (Karl Suss MA6). These mesas were then dry etched using a Deep Reactive Ion Etcher (DRIE; SPTS Pegasus). The etch rate (~50 nm/cycle) of the DRIE was validated by measuring the etch depth (as a function of time) using a profilometer (Bruker Dektak 150) scan. The DRIE etch was timed to stop at the BOX layer. Next, the wafer was submerged in BOE for approximately 2 hours in an orbital shaker (VWR® Scientific Standard Orbital Shaker, Model 5000) set to 70 rpm to etch the BOX layer and sacrificially release the particles from the Si handle substrate. When the microparticles were released from the silicon handle substrate, they were rinsed into a petri dish using deionized (DI) water. Next, a calibrated pipette was used to extract 5 μL of the microparticles and DI water. In a second petri dish, this 5 μL volume of water and the microparticles were mixed with 20 μL of Millipore deionized water. This mixed volume containing the microparticles was then dispensed into a small plastic Eppendorf vial. The vial was then centrifuged (4000 rpm for approximately 5 min, VWR® Scientific Centrifuge), the supernatant was discarded, and the pellet of microparticles was diluted with deionized water to a final volume of 30 μL. A 5 μL droplet of the suspended microparticles was then dispensed into the experimental liquid

chamber with electrodes for testing (further explained in the Methods Section of the main manuscript).

Supplementary Note 3: Fabrication and sacrificial release of n-type microparticles with one metal contact (N-I)

For the n-type microparticles with one metal contact (N-I), 4 μm x 4 μm square metal contacts were patterned using topside negative photolithography (JSR Micro NFR Negative Photoresist Series) and a mask aligner (Karl Suss MA6). The sample was then ashed in an oxygen (O_2) plasma (Emitech K-1050X) for good metal adhesion, and the metal contacts were vacuum deposited using electron-beam (e-beam) metal evaporation (Kurt Lesker PVD 75). The deposited contacts were Titanium (Ti; 800 Å)/Nickel (Ni; 600 Å)/Gold (Au; 2000 Å). The Ti layer served as a barrier metal to avoid the gold from diffusing into the silicon.[1] The negative photoresist was then lifted-off using a chemical solvent (Microposit® Remover 1165; Dow®). Next, the metal contacts were annealed using a rapid thermal annealer (Jipelec JetFirst 100 RTA) at 350°C for 10 sec to form a lower resistance contact and to improve adhesion of the contacts.

Next, the individual rectangular microparticle mesas were aligned to the metal contacts using fiducial markers and a mask aligner (Karl Suss MA6), patterned using topside positive photolithography (Microposit® S1813® Positive Photoresist), dry-etched using DRIE (SPTS Pegasus), wet-etched BOE), and released (BOE) from the substrate using the same process as described in Supplementary Note 2.

Supplementary Note 4: Fabrication and sacrificial release of p-n junction microparticles (PN-0)

To form a p-n junction, a p-type region was defined and diffused into the n-type silicon device layer. First, a 150 nm thermal oxide layer was grown by heating the wafer at 1000°C for 5 hours in a high-temperature dry oxidation furnace (Tempress 6304 4-Stack O_2 atmosphere). Next, the thermal SiO_2 was patterned and wet-etched (BOE) to form diffusion windows for the microparticles, as this SiO_2 layer served as a diffusion mask for the formation of the p-n junction. Next, the n-type silicon device layer was coated with a boron-doped spin-on-glass (SOG, Borosilicate Film (Filmtronics, Inc.), surface concentration, $C_{surface}$ = 6.57 x 10^{20} cm^{-3}) to diffuse p-type regions into the silicon device layer at the locations of the etched oxide window areas. The SOG was annealed at 1050°C for 17.5 hours in a high-temperature (N_2 and O_2 atmosphere) furnace (Tempress 6304 4-Stack) to achieve a targeted 3.5 μm junction diffusion depth. Once the annealing process was complete, the residual dopant oxide and the thermal oxide diffusion mask were removed using a reactive ion etcher (Trion Technology Phantom II RIE; conditions: Pressure: 1500 mT, Power: 25 W RIE and ICP: 500 W for 5 min). A polymer resist etch (Trion Technology Phantom II RIE; conditions: Pressure: 500 mT, Power: 25 W RIE, ICP: 500 W and 50 sccm O_2 for 3 min), BOE submersion (until the surface became hydrophobic) and a piranha clean (3:1 volume ratio of H_2SO_4:H_2O_2 at 105°C for 10 min) were performed to ensure that any residual oxides were fully removed.

Next, the individual rectangular mesas were patterned, dry-etched, wet etched, and released from the substrate using the same process as Supplementary Note 2.

Note that the fiducial markers on the masks for the p-type dopant ensured that the mesas were aligned with the doped regions to form a lateral p-n junction.

Supplementary Note 5: Fabrication and sacrificial release of p-n junction microparticles with one metal contact (PN-I) and p-n junction diode microparticles with two metal contacts (PN-II)

For the p-n junction microparticles with one metal on the n-side of the junction, first, the p-n junctions were lithographically defined as described in Supplementary Note 4. Next, the metal contact (single) or contacts (two) were aligned, patterned, deposited, and annealed as described in Supplementary Note 3. Then, the individual rectangular mesas were aligned, patterned, dry-etched, wet etched, and released from the substrate using the same processes as described in Supplementary Note 2.

The images in Supplementary Figure 1 show microparticles at different optical magnifications. These images include photomicrographs of n-type microparticles without contacts (Supplementary Figure 1a), photomicrographs of p-n junction microparticles without metal contacts (Supplementary Figure 1b), a scanning electron microscope (SEM) image of a p-n junction microparticle (Supplementary Figure 1c), two photomicrographs of n-type microparticles with one metal contact (Supplementary Figure 1d and Supplementary Figure 1e), and photomicrographs of p-n junction diode microparticles (Supplementary Figure 1f), in all cases, after the DRIE mesa etch process was completed. Supplementary Figure 1a-b highlight the monodisperse nature of the particles, which is common for high-volume semiconductor microfabrication

processing, and Supplementary Figure 1d, in particular, emphasizes that many monodisperse particles can be fabricated in a single wafer run.

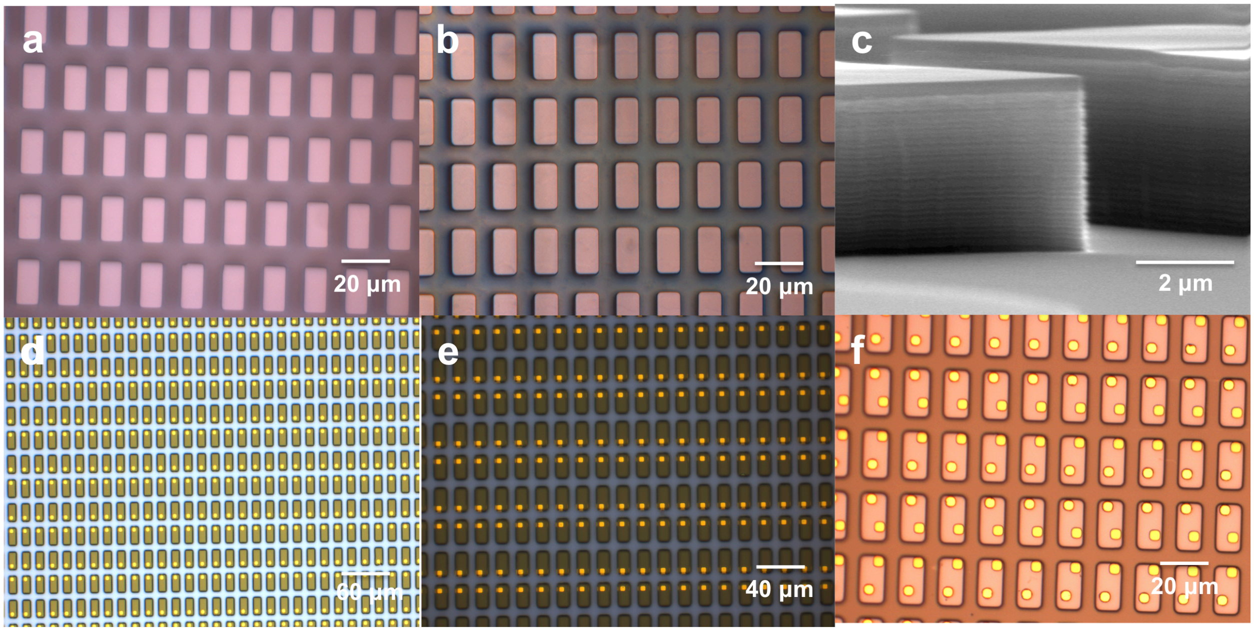

Supplementary Figure 1| Images of the n-type silicon microparticles and p-n junction diode microparticles before release from the SOI substrate:**(a)** photomicrograph of n-type microparticles without contacts (N-0); **(b)** p-n junction microparticles without metal contacts (PN-0); **(c)** scanning electron images (FEI XL30 SEM-FEG) of p-n junction microparticles without metal contacts (PN-0); **(d,e)** photomicrographs of n-type microparticles with one metal contact (N-I). **(f)** photomicrographs of p-n junction diode microparticles with two metal contacts (PN-II)

After various points throughout the chemical release process, photomicrographs were taken of n-type microparticles (Supplementary Figure 2a), p-n junction microparticles with one metal contact (Supplementary Figure 2b) and p-n junction diode microparticles with two metal contacts (Supplementary Figure 2c). Supplementary Figure 2d is a photomicrograph of released microparticles on a glass slide (VWR® Scientific) underneath a plastic cover slip (VWR® Scientific).

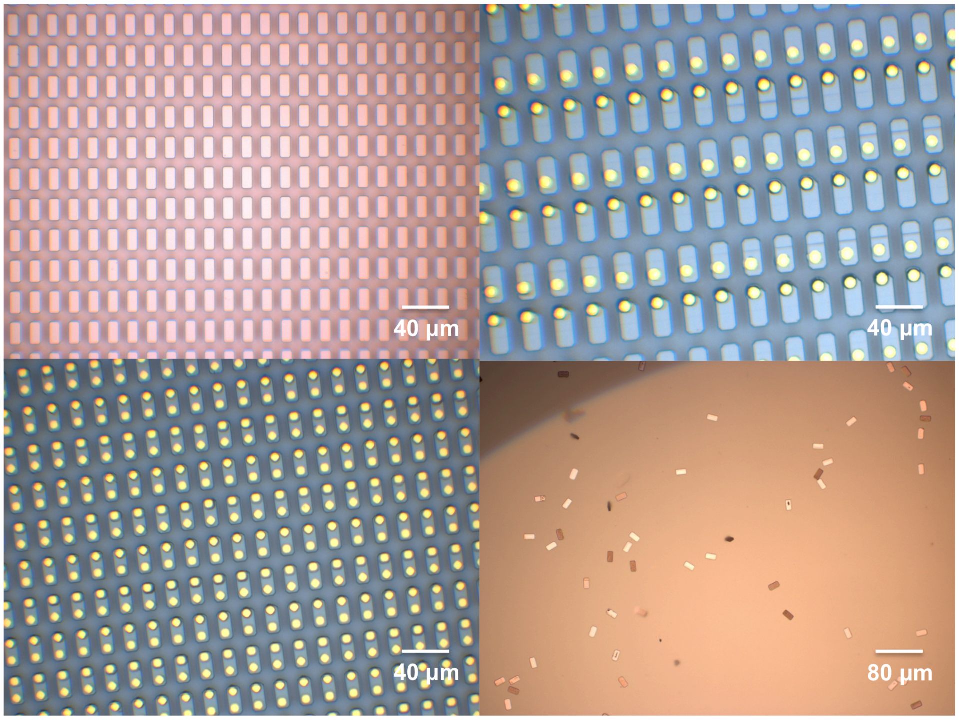

Supplementary Figure 2| Photomicrographs of n-type silicon microparticle and p-n junction diode microparticles. (a) Photomicrograph of n-type microparticles after 5000 sec of submersion in BOE. The different oxide colors between the microparticles and diode microparticles indicate different etched oxide thicknesses[2] **(b)** Photomicrograph of p-n junction microparticles with one metal contact after 6000 sec of submersion in BOE; **(c)** Photomicrograph of partially released p-n junction diode microparticles with two metal contacts; **(d)** Photomicrograph of microparticles released from the SOI substrate and suspended in water on a glass slide.

Supplementary Note 6: Characterization of n-type silicon microparticles with one metal contact (N-I) and p-n junction diode microparticles with two contacts (PN-II)

To characterize the p-type and n-type semiconductor-metal interfaces, prior to mesa etching and sacrificially releasing from the handle substrate, a representative subset of n-type microparticles with one contact (N-I) and p-n junction diode microparticles with two metal contacts (PN-II) were electrically probed using a Keithley 4200 Source Measurement Unit (SMU) to acquire a current-voltage (I-V) characteristic curve. In all cases, two 200 nm diameter, 2” long Tungsten Cat Whisker electrical probe tips (Lucas Signatone, Corp.) were used to probe the metal contacts on the devices, as shown in Supplementary Figure 3a. The resulting I-V characteristic curve for the p-n microdiode with probes on the n and p sides of the device is shown in Figure 5b of the main manuscript. For the N-I microparticles, the two probes were used to contact n to n (metal on n-side of the particle), with the results shown in Supplementary Figure 3b. The n-n ohmic measurement (probing between n-ohmic metal contacts on two neighboring microparticles) yielded a resistive I-V curve. This is due to the (i) low doping concentration of the n-type semiconductor at the n-semiconductor-metal interface and the (ii) ohmic metal (Ti/Ni/Au) interfacing with the low-doped n-type semiconductor.

For the PN-II diode microparticles, the two probes were used to contact p to p (metal on the p-side of the diode; p-p) and n to n (metal on the n-side of the diode; n-n), with the results shown in Supplementary Figure 3c. The n-n ohmic measurement (probing between n-ohmic metal contacts on two neighboring diode microparticles) yielded an I-V curve that was more resistive than the same p-p measurement (probing between p-ohmic metal contacts on two neighboring microparticles). This is due to the

low doping concentration of the n-type semiconductor at the n-semiconductor-metal interface compared to the higher p-type diffused doping concentration at the p-semiconductor-metal interface.

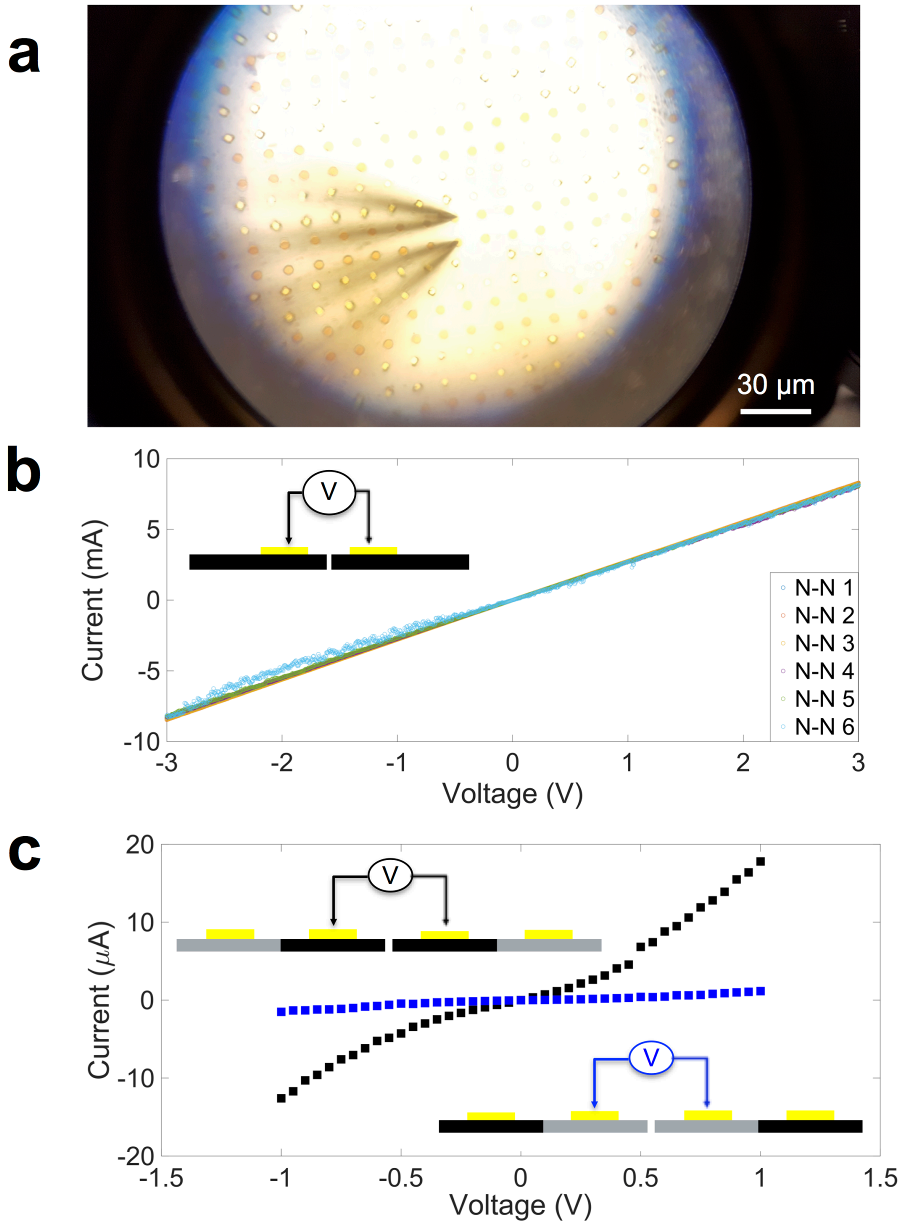

Supplementary Figure 3| Characterization of p-n junction diode microparticles (PN-II). (a) Photomicrograph of two tungsten cat whisker probes touching two 4 μm x 4 μm adjacent metal contacts. **(b)** I-V characteristic curves between several n-to-n metal contacts on two neighboring microparticles. **(c)** I-V characteristic curves between n-to-n metal contacts on two neighboring diode microparticles (black curve) and between p-to-p metal contacts on two neighboring diode microparticles (blue curve).

Supplementary Note 7: Tracking analysis of active self-propelling silicon microparticles

All microparticles velocities were tracked using Fiji, an image-processing package based on ImageJ (NIH). Each experiment captured a minimum of five microparticles that were tracked by the software. We only considered microparticles where the metal contacts were facing up. We classified the microparticles into two different groups depending on their propulsion mechanism: (i) microparticles with one metal contact (i.e., n-type microparticles with one metal contact N-I, p-n junction microparticles with one metal contact on the n-side of the p-n junction; PN-I, and p-n junction diode microparticles with one metal contact on the p-side of the p-n junction; PN-I) and (ii) microparticles with zero or two symmetrical metal contacts (i.e., n-type microparticles and p-n junction microparticles without any metal contacts; N-0 and PN-0, and p-n junction diode microparticles with two metal contacts; PN-II).

First, microparticles with symmetrical metal contacts (N-0, PN-0, and PN-II) exhibited frequency-independent behavior. The reason for this relationship is that the microparticles (i) were weakly polarized or (ii) rectify the external AC electric field into a local DC potential. For the N-0 case, the uniform doping concentration (N_D is approximately equal to 10^{15} cm^{-3}) created frequency-independent behavior across the microparticle. For the PN-0 case, the non-uniform doping concentration (N_D is approximately equal to 10^{15} cm^{-3} on n-side; N_A is approximately equal to 10^{20} cm^{-3} on the p-side surface and follows complementary error function (erfc) profile towards the bottom of the particle) created frequency-independent behavior across the microparticle. For the PN-II case, the AC electric field is rectified during the positive half of the cycle and converts this energy into a local DC field potential. This DC field

creates a self-electroosmotic flow around the microparticles, leading to powered self-propulsion by driving the induced negative charges in the electric double layer into the cathode of the microdiodes.[10] The DC electroosmotic velocity can be derived using the Helmholtz-Smoluchowski equation:[11]

$$v_{eo} = -\frac{\varepsilon_m \varepsilon_0 \zeta}{\mu} E_{applied} \text{ (Supplementary Equation 1)}$$

where v_{eo} is the electroosmotic velocity, ε_0 is the dielectric permittivity of vacuum, ε_m is the dielectric permittivity of the medium, ζ is the induced zeta potential at the surface of the microparticle, μ is the dynamic viscosity of the medium and $E_{applied}$ is the applied AC electric field.[10] The velocity across the n-type microparticle (N-0) and the p-n junction microparticle (PN-0) can be expressed by:

$$v_{n-type} = \beta \frac{\varepsilon_m \varepsilon_0 \zeta}{2\mu} E_{applied} \text{ (Supplementary Equation 2)}$$

where β is the hydrodynamic-resistance correction factor. The velocity across the p-n junction diode microparticle with two metal contacts can be expressed by:

$$v_{diode} = \beta \frac{\varepsilon_m \varepsilon_0 \zeta}{2\mu} \left(E_{applied} - E_{d0}\right) \text{ (Supplementary Equation 3)}$$

where E_{d0} is the electric field across the diode microparticle. Supplementary Equation 1 and Supplementary Equation 2 and Supplementary Equation 3 indicate that the velocity of the microparticles with symmetrical contacts is dependent on the applied AC electric field, but independent on the frequency of the field. Supplementary Figure 4a illustrates the field-strength dependent phenomenon across microparticles with symmetric contacts. Supplementary Figure 4b illustrates the frequency-independent phenomenon across microparticles with symmetrical contacts.

Next, the p-n junction microparticles with one metal contact exhibited velocity dependence as a function of the electric field magnitude squared (E^2). For the PN-I

case, the average velocity data as a function of E^2 is shown in Supplementary Figure 4c Given the similarity in velocities of both types of PN-I microparticles, it is apparent that their motions are dominated by a combination of the strong-ICEP effects (at low AC electric field frequencies) from the metal contact and DEP effects (high AC electric field frequencies) from dipolar interactions. While the non-uniform doping concentration (weak-ICEP) across the p-n junction is also present, the strong-ICEP effects from the metal still dominated the propelling direction of the particle.

The microparticles with one metal contact exhibited distinct behaviors depending on the frequency of the externally applied AC electric field.[6-7] At low frequencies (i.e., less than 10 kHz), a nonlinear distribution of induced charges formed on the surface of the microparticles, across the microparticles, and created an imbalanced electroosmotic flow, contributing to microparticle powered propulsion.[6-7] When the electric field was turned on, the electric double layer on the metallized side of the microparticles was more strongly polarized than the non-metallized side. Consequently, a stronger induced-charge electroosmotic (ICEO) slip was formed around the metal region and the induced-charge electrophoretic (ICEP) force propelled the microparticles with the metal contact as the propeller. At high frequencies (i.e., greater than or equal to 10 kHz), dielectrophoretic forces were dominant, causing the microparticles to self-assemble and form linear chains.[8] In this frequency regime, AC-electrohydrodynamic (AC-EHD) flow was negligible and the dominant fluid flow was due to electrothermal effects.[9] The effect can be explained by referring to the Stern layer developed across the fluid.[9] The Stern layer can be modeled as a fixed electrolytic capacitor between the cell electrodes.[9] At high ionic conductivities (e.g., at high electric field frequencies), the diffuse layer of the

electrolytic capacitor was negligible and a large potential drop existed across the Stern layer. Since the microparticle velocity was proportional to the free charge across the diffuse layer, AC-EHD flow decreased and the overall velocity of the microparticle decreased. Supplementary Figure 4d illustrates the two frequency regimes of the asymmetrically polarized n-type (N-I) and p-n junction microparticles (PN-I; one metal contact on n-side of junction or one metal contact on p-side of junction).

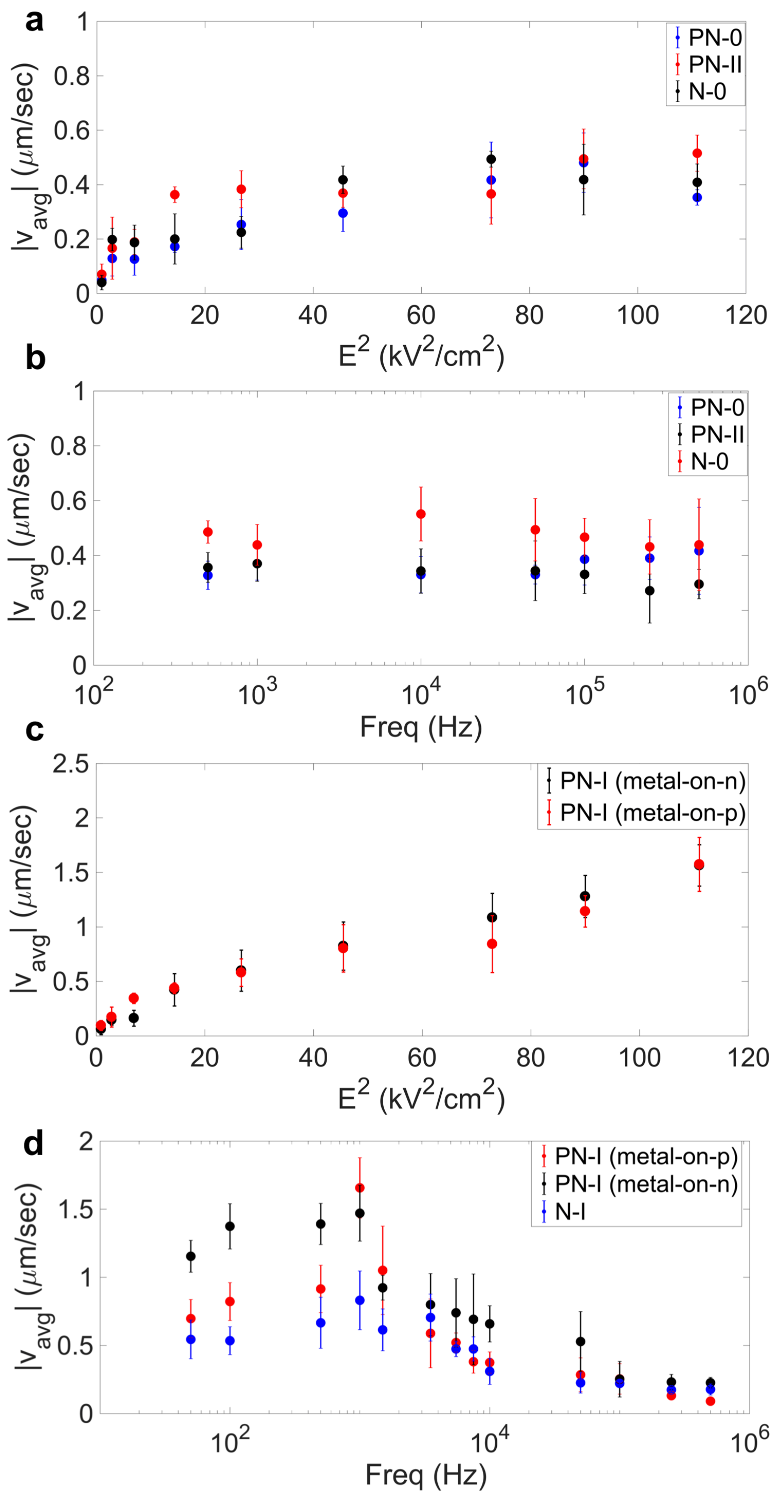

Supplementary Figure 4| Tracking analysis of active silicon microparticles. (a) Tracking analysis of microparticles with symmetrical metal contact(s) (i.e., zero or two metal contacts; including N-0, PN-0, and PN-II). These velocities show a second-order effect as a function of the electric field magnitude squared (100 Hz). **(b)** Tracking

analysis of microparticles with symmetrical metal contact(s) (i.e., zero or two metal contacts; including N-0, PN-0, and PN-II). These microparticle compositions show velocity independence as a function of the applied AC frequency. The AC electric field strength was fixed at 54.4 kV^2 cm^{-2}. **(c)** Tracking analysis of microparticles with one metal contact (i.e., N-I and PN-I). These velocities show a first-order effect as a function of the electric field magnitude squared (at 100 Hz). **(d)** Tracking analysis of microparticles with an asymmetrical metal contact (i.e., one metal contact; including N-I and PN-I (one metal contact on n-side of p-n junction or one metal contact on p-side of p-n junction). This data shows velocity dependence as a function of the applied AC frequency. The AC electric field strength was fixed at 54.4 kV^2 cm^{-2}. Each data point represents the average and standard deviation (one above and one below for the error bars), as measured from five different microparticles in a single experiment.

Supplementary Note 8: Hydrodynamic analysis of N-0 and PN-II microparticles

500 nm 4% w/v_polystyrene spheres used as tracers (Thermo Fisher Scientific Inc.) were suspended in a dilute concentration of active self-propelling silicon particles. All tracer particle experiments were processed using PIVlab, a time-resolved digital particle image velocimetry graphical user interface tool developed in MATLAB (MathWorks, Inc.) The original images from the tracer experiments were fragmented in ImageJ (NIH) and processed in PIVlab. A microparticle mask was set to calculate the hydrodynamic flows and velocity characteristics around the particles. The radius of the tracer particles set the interrogation area. A direct Fourier transform correlation with multiple passes and deforming windows was used to produce a higher signal-to-noise ratio and a robust cross-correlation calculation. A calibration (pixels/meter and time step between images) was set before the analysis was performed. A time-resolved movie and x-/y-velocity characteristics, for each particle composition, were exported from PIVlab, imported into MATLAB, and plotted in MATLAB.

Tracer particle experiments (E^2 = 54.4 kV^2 cm^{-2} and 500 Hz; Supplementary Movie 17 and Supplementary Movie 18) were performed to visualize the relationship

between the isotropic polarizability, anisotropic polarizability, and the DC propulsion of the powered microparticles. Beads were suspended in the chamber and encircled the microparticles. For the case of the PN-II diode microparticles, the tracer particles slowly flowed towards the p-n junction of the particles (Supplementary Movie 17 and Supplementary Figure 5a-b). The tracer particles propelled at an average net velocity of 400 nm/sec in the x-direction (Supplementary Figure 5c). For the case of the N-0 microparticles, the tracer particles flowed away from the microparticles with an intermediate speed (Supplementary Movie 18 and Supplementary Figure 5d-e). The tracer particles propelled at an average net velocity of 600 nm/sec in the x- direction (Supplementary Figure 5f).

For the PN-II case, there was non-uniform doping concentration (N_d is approximately equal to 10^{15} cm^{-3} on n-side, and N_a is approximately equal to 10^{20} cm^{-3} on p-side surface, with an erfc profile toward the bottom of particle). There were ohmic metal contacts (Ti/Ni/Au on n-side; Al/Ti/Ni/Au on p-side) on both sides of the p-n junction that competitively balanced the flows on both sides of the microparticle. For the N-0 case, the uniform doping concentration across the microparticle caused the particle to propel mostly by bulk flow. Overall, these results indicated that symmetrically polarized surfaces significantly impair the microparticle propulsion, suggesting that we can program active modes of propulsion by adjusting the relative contribution of flows resulting from each mechanism.

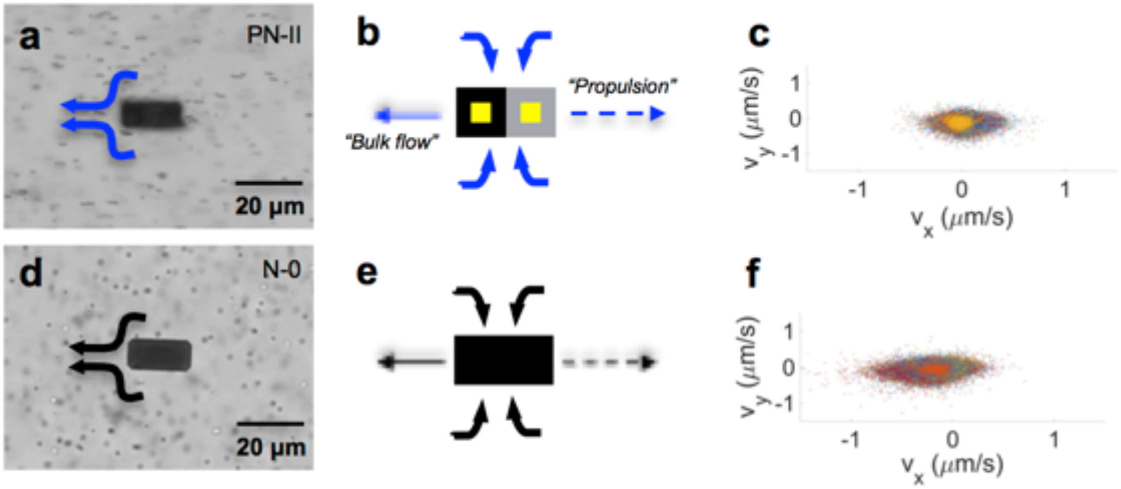

Supplementary Figure 5| Hydrodynamic analysis of PN-II and N-0 type microparticles. a-b. Slow hydrodynamic flows around a PN-II diode microparticle, indicating weak anisotropic polarization from the balance of two metal contacts on the microparticle. **c.** x- and y-velocity distribution of tracer particles surrounding the PN-II particle. **d-e.** Slow hydrodynamic flows around an N-0 microparticle, indicating isotropic polarization from the uniform doping concentration across the microparticle. **f.** x- and y-velocity distribution of tracer particles surrounding the N-0 particle. All experiments are shown and plotted at a fixed electric field strength (E^2 = 54.4 kV^2 cm^{-2}) and frequency (500 Hz).

Supplementary Note 9: Collective phenomena of self-propelling active microparticles (with tracer particles): Reversible assembly of PN-0 and PN-I microparticles

Particles with a 500 nm diameter (4% w/v_polystyrene; Thermo Fisher Scientific Inc.) were suspended in a dilute concentration of silicon microparticles and used as tracer particles. Tracer particle experiments (E^2 = 54.4 kV^2 cm^{-2}; Supplementary Movie 19 and Supplementary Movie 20), using a collective number of microparticles were performed to visualize the relationship between the anisotropic polarizability and the ICEP propulsion of the microparticles.

For the PN-0 case (Supplementary Movie 19), the microparticles disassembled (Supplementary Figure 6a, Supplementary Figure 6c, and Supplementary Figure 6e) and propelled at low electric field frequencies (500 Hz). At this frequency, encircling

tracer particles actively flowed towards the p-n junction region of the microparticle, the polarizability from the ionic layer around the p-n junction decreased, and weak anisotropic forces (weak-ICEP from non-uniform doping concentration) were dominant. At high electric field frequencies (500 kHz), the encircling tracer particles passively flowed towards the self-propelling PN-0 microparticle, the polarizability from the ionic layer around the PN-0 microparticle increased, and the PN-0 microparticles assembled (Supplementary Figure 6b, Supplementary Figure 6d, and Supplementary Figure 6f) by dipolar polarization forces. The encircling tracer particles passively flowed towards the self-propelling particle.

For the PN-I case (Supplementary Movie 20), the microparticles disassembled (Supplementary Figure 7a, Supplementary Figure 7c, and Supplementary Figure 7e) and propelled at low electric field frequencies (500 Hz). At this frequency, encircling tracer particles actively flowed towards the p-n junction and metal region of the microparticle, the polarizability from the ionic layer around the metal contact and the p-n junction decreased, and strong anisotropic forces (strong-ICEP from highly polarizable metal) were dominant. At high electric field frequencies, the encircling tracer particles passively flowed towards the metal contact and the PN-I microparticle, the polarizability from the ionic layer around the metal contact and the PN-I microparticle increased, and the PN-I microparticles assembled (Supplementary Figure 7b, Supplementary Figure 7d, and Supplementary Figure 7f) by dipolar polarization forces.

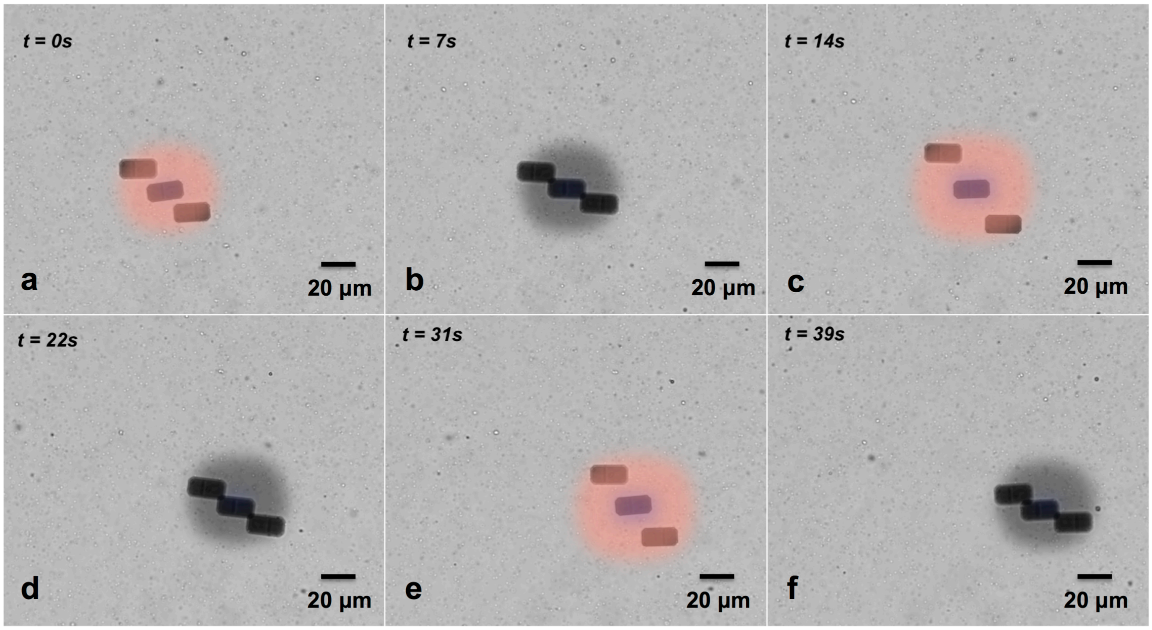

Supplementary Figure 6| Reversible assembly of PN-0 microparticles (with encircling tracer particles). a, c, and e. At low electric field frequencies (500 Hz), the microparticles disassembled by weak anisotropic polarization forces (non-uniform doping concentration across particle). For this frequency case, the tracer particles flowed towards the p-n junction. **b, d, and f.** At high electric field frequencies (500 kHz), the microparticles assembled by dipolar polarization forces. For this frequency, the tracer particles passively flowed towards particle.

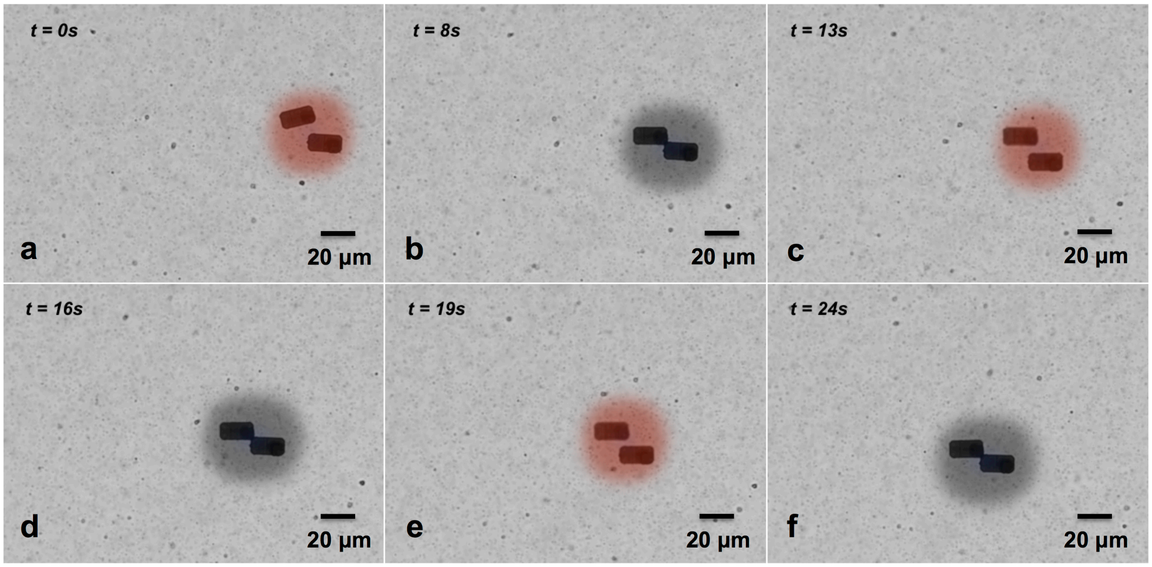

Supplementary Figure 7| Reversible assembly of PN-I microparticles (with encircling tracer particles). a, c, and e. At low electric field frequencies (500 Hz), the microparticles disassembled by strong anisotropic polarization forces (highly polarizable metal contact and weak anisotropy stemming from non-uniform doping concentration).

For this frequency, the tracer particles flowed towards the p-n junction. **b, d, and f.** At high electric field frequencies (500 kHz), the microparticles assembled by dipolar polarization forces. For this frequency, the tracer particles passively flowed towards particle.

Supplementary Note 10: Collective phenomena of self-propelling active microparticles: Dielectrophoretic chaining at high electric field frequencies (≥ 10 kHz)

One of the attractive features of silicon microparticles was the ability to inexpensively fabricate millions of monodisperse or intentionally multidisperse particles in a single fabrication run. When studied at varying concentrations, the microparticles assembled into a variety of architectures, as can be seen in Supplementary Figure 8. For the case of 2 self-propelling microparticles (Supplementary Movie 14 and Supplementary Figures 8a-b), doublet structures were formed at high electric field frequencies. For the case of about 50-100 self-propelling microparticles (Supplementary Movie 15 and Supplementary Figures 8c-d), staggered or linear chain structures were formed at high electric field frequencies. For the case of about several 100 -1000 self-propelling microparticles (Supplementary Movie 16 and Supplementary Figures 8e-f), “traffic-jamming” structures were formed at high electric field frequencies.

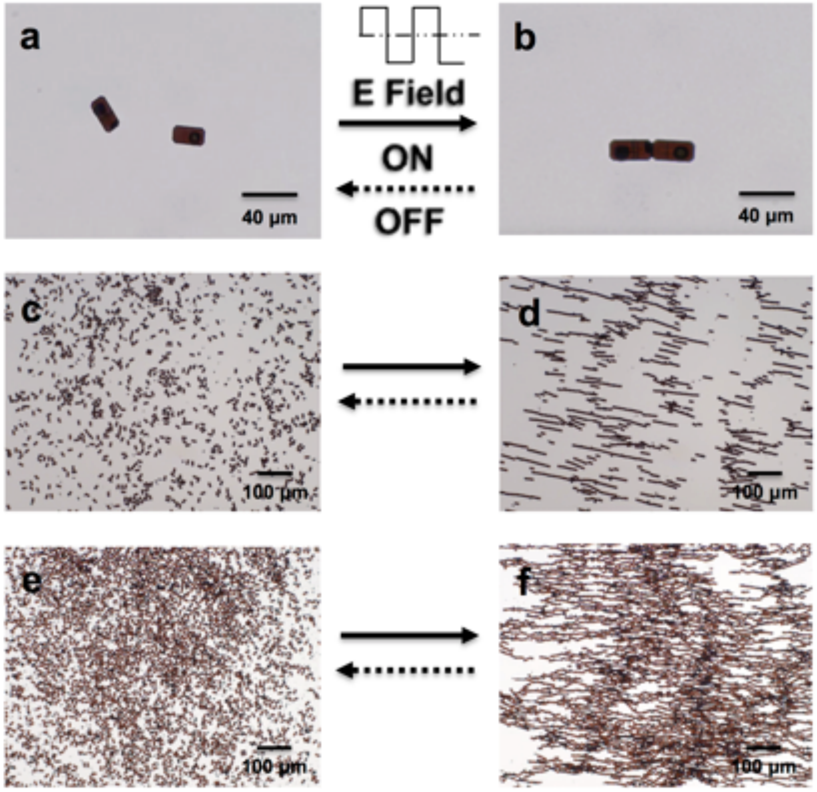

Supplementary Figure 8| Directional assembly and programmable disassembly of PN-I type microparticles. (a-b) Micrographs of PN-I microparticles assembling into a doublet. At low AC electric field frequencies, the highly polarized metal propels the particles towards each other. **(c-d)** Photomicrographs of hundreds of microparticles assembling into many linear chains. **(e-f)** Photomicrographs of many hundreds of microparticles assembling into longer chains. The arrows between sets of images indicate that the assembled structures can be rapidly and intentionally disassembled by decreasing the frequency below a certain threshold (< 10 kHz).

Supplementary Note 11: Electrostatic potential distribution across various key interfaces

Many semiconductors (e.g., silicon) form a thin SiO_2 layer when immersed in water. This oxide layer provides positive and negative chemical groups (such that hydrogen ions can more easily attract to, or repel from, the surface) that are polarized when exposed to an applied external electric field in water.[3]

The density of most surface charges can be solved using the Poisson equation:

$$\nabla^2\psi = -\frac{\rho}{\varepsilon_r\varepsilon_0} \text{ (Supplementary Equation 4)}$$

where ρ represents the charge density, ψ represents the electrostatic potential, ε_r represents the relative dielectric permittivity of the water medium and ε_0 represents the relative dielectric permittivity of vacuum. The charge density for each material layer can be described using the following equations:[3-4]

$$\rho_{silicon} = N_C\mathcal{F}_{1/2}(\eta_F) \text{ (Supplementary Equation 5)}$$

$$\rho_{metal} = I/Aqv_d \text{ (Supplementary Equation 6)}$$

$$\rho_{oxide} = 0 \text{ (Supplementary Equation 7)}$$

$$\rho_{water} = \sum_i n_i^0 z_i exp\left(\frac{-z_i e\psi}{k_B T}\right) \text{ (Supplementary Equation 8)}$$

where N_c is the conduction effective density of states, $\mathcal{F}_{1/2}(\eta_F)$ is the Fermi integral of ½, which is the probability of energy level occupation by an electron.[5] The effective density of states for electrons in the conduction band and the Fermi integral can be expanded using the following equations:

$$N_C = \left(\frac{2(2\pi)m_n^* k_B T}{h^3}\right)^{3/2} \text{ (Supplementary Equation 9)}$$

$$\mathcal{F}_{1/2}(E) = \frac{2}{\sqrt{\pi}}\int_0^\infty \frac{E^{1/2}}{1+exp(E-E_F)} \text{ (Supplementary Equation 10)}$$

where m_n^* is the effective mass for an electron, $k_B T$ is the product of the Boltzmann constant and temperature, h is the Planck constant, E is the energy at which the function is evaluated, and E_F is the energy of the Fermi level. For the charge density of a metal, the variable I represents the current flowing through the conductor, A represents the cross-sectional area of the conductor, q is the electron charge, and v_d is the electron drift velocity. For the charge density of water, n_i is the concentration of the

i^{th} ionic component and z_i is the number of valence electrons of the ith ionic component in the fluid.

The density surface charges at the oxide-water interface can be represented by the difference between the positive and negative surface charge densities.[3] The surface charge density is linearly related to the electrostatic potential gradient and is given by:

$$\sigma_{surface} = -\varepsilon_r \varepsilon_0 \nabla\psi \cdot \boldsymbol{n} \text{ (Supplementary Equation 11)}$$

where $\boldsymbol{n}$ is the vector normal pointing to the electrolyte interface. The surface potential was estimated by placing a linear fit on the velocity data as a function of the voltage gradient. The slope of the line was used to measure the surface potential.

The polarizability of the microparticles strongly affects the powered propulsion of the particle. For example, the p-n junction microparticles were more polarizable than the n-type particle. Adding an asymmetric metal contact to the microparticles introduced a metal-semiconductor junction interface and increased the polarizability of the particles; however, adding two symmetric contacts decreased the polarizability of the particles. The least polarizable microparticle was the n-type microparticle without a metal contact (N-0). When a metal contact was deposited on one-half of the n-type material, the polarizability increased due to the metal, the interface between the metal and the semiconductor material, and a higher particle velocity was measured. The n-type microparticle with one contact (N-I) was more polarizable than the p-n junction microparticle without metal contacts (PN-0). However, the p-n junction microparticles with one metal contact on either side of the device (PN-I) had the highest polarizability of all of the morphologies studied, as well as the highest velocity. Finally, the p-n

junction diode microparticle with two symmetric contacts (PN-II) was comparable in polarizability to the PN-0 microparticle.

Image tracking analysis was performed to analytically estimate the electrostatic potential of the n-type microparticles, p-n junction microparticles, and p-n junction diode microparticles. The microparticles propelled in response to an applied AC electric field (ranging from 0.9 kV^2 cm^{-2} - 111 kV^2 cm^{-2}) and AC frequency (ranging from 50 Hz – 500 kHz). The average velocity of each microparticle was collected and linearly fitted as a function of the applied electric field strength. The overall surface potential for all of the microparticles (and their respective swimming directions) was estimated from the slope of the linear fit using Supplementary Equation 11 and summarized in Supplementary Table 1.

Supplementary Table 1| Estimated surface potentials for various silicon microparticles using Supplementary Equation 11. The black regions illustrate the n-type silicon (electrons as the majority carrier); light grey regions illustrate the p-type silicon (holes as the majority carrier); gold regions illustrate the metal contacts on both sides of the p-n junction. The black arrows illustrate the propulsion direction of the microparticles.

Si Particle Morphology	*Estimated Surface Potential*
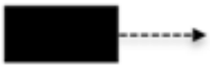	11 mV
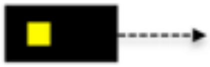	19 mV
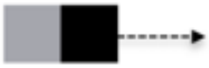	13 mV
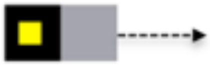	64 mV
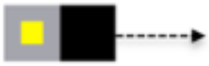	43 mV
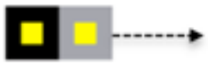	12 mV

Supplementary References

1. Jaeger, R. C. *Introduction to Microelectronic Fabrication, Second Edition*. (Prentice Hall, Upper Saddle River, 2002).
2. Henrie, J., Kellis, S., Schultz, S. & Hawkins, A. Electronic color charts for dielectric films on silicon. *Opt. Express* **12,** 1464–1469 (2004).
3. Fleharty, M. E., van Swol, F. & Petsev, D. N. Charge regulation at semiconductor-electrolyte interfaces. *J. Colloid Interface Sci.* **449,** 409–415 (2014).
4. Lew, H. S., Yon, K. L., & Hyon, S. L. Convective electric current flowmeter. *U.S. Patent*. **5,247,836**. (1995).
5. Feynman, R. P., Metropolis, N. & Teller, E. Equations of state of elements based on the generalized fermi-thomas theory. *Physical Review* **75,** 1561–1573 (1949).
6. Squires, T. M. & Bazant, M. Z. Breaking symmetries in induced-charge electro-osmosis and electrophoresis. *Journal of Fluid Mechanics* **560,** (2006).
7. Gangwal, S., Cayre, O. J., Bazant, M. Z. & Velev, O. D. Induced-charge Electrophoresis of Metallodielectric Microparticles. *Phys. Rev. Lett.* **100**, 058302 (2008).
8. Gangwal, S., Cayre, O. J. & Velev, O. D. Dielectrophoretic assembly of metallodielectric Janus particles in AC electric fields. *Langmuir* **24**, 13312-13320 (2008).
9. Ramos, a, Morgan, H., Green, N. G. & Castellanos, A. AC electrokinetics: a review of forces in microelectrode structures. *J. Phys. D. Appl. Phys.* **31,** 2338–2353 (1999).
10. Chang, S. T., Paunov, V. N., Petsev, D. N. & Velev, O. D. Remotely powered self-propelling microparticles and micropumps based on miniature microdiodes. *Nat. Mater.* **6,** 235–240 (2007).
11. Park, H. M. & Lee, W. M. Helmholtz-Smoluchowski velocity for viscoelastic electroosmotic flows. *J. Colloid Interface Sci.* **317,** 631–636 (2008).
