# Supplementary material for: Reconfigurable engineered motile semiconductor microparticles
Source: Nat Commun. 2018 May 3;9:1791. doi: 10.1038/s41467-018-04183-y (PMC5934469; doi:10.1038/s41467-018-04183-y)
Supplement: Supplementary file 2 — Description of Additional Supplementary Files [file 41467_2018_4183_MOESM2_ESM.docx]

**Description of Additional Supplementary Files**

File Name: Supplementary Movie 1

Description: Hydrodynamic Experiments of Singular PN-0 Microparticle (70 Vpp, 500 Hz)

File Name: Supplementary Movie 2

Description: Hydrodynamic Experiments of Singular N-I Microparticle (70 Vpp, 500 Hz)

File Name: Supplementary Movie 3

Description: Hydrodynamic Experiments of Collective N-I Microparticles (70 Vpp, 500 Hz)

File Name: Supplementary Movie 4

Description: Hydrodynamic Experiments of Singular PN-I Microparticle (70 Vpp, 500 Hz)

File Name: Supplementary Movie 5

Description: Hydrodynamic Experiments of Collective PN-I Microparticles (70 Vpp, 500 Hz)

File Name: Supplementary Movie 6

Description: Synchronized Propulsion & Repulsion of N-I Microparticles I (70 Vpp, 100 Hz)

File Name: Supplementary Movie 7

Description: Synchronized Propulsion & Repulsion of N-I Microparticles II (70 Vpp, 100 Hz)

File Name: Supplementary Movie 8

Description: Synchronized Propulsion & Repulsion of PN-0 Microparticles (70 Vpp, 100 Hz)

File Name: Supplementary Movie 9

Description: Reversible Assembly of N-I Microparticles (50 Vpp, 100 Hz <-> 100 kHz)

File Name: Supplementary Movie 10

Description: Leftward Directional Propulsion of PN-0 Microparticle (70 Vpp, 500 Hz)

File Name: Supplementary Movie 11

Description: Rightward Directional Propulsion of PN-0 Micropartice (70 Vpp, 500 Hz)

File Name: Supplementary Movie 12

Description: Collective Reversible Assembly of PN-0 Microparticle (40 Vpp, 100 Hz <-> 1 kHz)

File Name: Supplementary Movie 13

Description: Collective Reversible Assembly of PN-I Microparticle (40 Vpp, 100 Hz <-> 1 kHz)

File Name: Supplementary Movie 14

Description: Induced Dipolar Polarization of PN-I Microparticles I (70 Vpp, 10 kHz)

File Name: Supplementary Movie 15

Description: Induced Dipolar Polarization of PN-I Microparticles II (70 Vpp, 10 kHz) 3

File Name: Supplementary Movie 16

Description: Induced Dipolar Polarization of PN-I Microparticles III (70 Vpp, 10 kHz)

File Name: Supplementary Movie 17

Description: Hydrodynamic Experiments of Singular PN-II Microparticle (70 Vpp, 500 Hz)

File Name: Supplementary Movie 18

Description: Hydrodynamic Experiments of Singular N-0 Microparticle (70 Vpp, 500 Hz)

File Name: Supplementary Movie 19

Description: Hydrodynamic Collective Experiments of Reversibly Assembled PN0 Microparticle (55 Vpp, 500 Hz <-> 500 kHz)

File Name: Supplementary Movie 20

Description: Hydrodynamic Collective Experiments of Reversibly Assembled PN-I Microparticle (55 Vpp, 500 Hz <-> 500 kHz)
